# New method for transient cold wall heat flux measurements for unlimited exposure times

**DOI:** 10.1038/s41598-025-03245-8

**Published:** 2025-05-29

**Authors:** Fabian Hufgard, Christian Duernhofer, Clemens Mueller, Stefan Loehle

**Affiliations:** High Enthalpy Flow Diagnostics Group (HEFDiG), Institute of Space Systems, University of Stuttgart, Pfaffenwaldring 29, 70569 Stuttgart, Germany

**Keywords:** Transient heat flux measurement, Inverse problem, Aerospace engineering, Characterization and analytical techniques

## Abstract

This paper presents a new method to measure high cold wall surface heat fluxes temporally resolved. It is based on a system identification approach, the so-called Non-Integer System Identification (NISI) method, which allows to correlate the surface heat flux to one single in-depth temperature measurement. Here, the model parameters are found experimentally by non-destructive calibration of the actual sensor hardware. The key feature of the new method is that the sensing wall segment is actively water-cooled at the backside, so that a transient, i.e. temporally varying, heat flux can be determined over long periods of time without damaging the sensing element. In the paper, the NISI model equation is derived from the heat equation considering this cooled backside boundary condition. We prove the useful applicability of our new approach by assessing data from a numerical thermal simulation. The determined heat flux shows excellent agreement with the known input data.

## Introduction

Accurate measurement of transient surface heat flux is crucial for assessing the performance of thermal protection systems, whether in combustion chambers or re-entry spacecraft^1^. Although there are a number of measurement methods available^2,3^, current techniques for temporally resolved measurements are limited by comparably short measurement durations, an inability to resolve unsteady conditions under realistic thermal boundary constraints, or by significantly altering the state of the sensor surface.

This paper introduces a novel method for the continuous measurement of transient surface heat flux onto a cold copper wall. The method achieves theoretically infinite measurement times by actively cooling the backside of the sensor, thereby enabling reliable operation even under sustained high heat loads. A sensor based on this principle supports at least two critical application scenarios: (1) characterization of transient plasma flow conditions in ground testing facilities for atmospheric re-entry flight, and (2) heat flux measurements inside combustion chambers of rocket engines.

Re-entering spacecraft are either meant to reach the Earth’s surface safely, e.g. return capsules, or demise in the Earth’s atmosphere. Return capsules require a thermal protection system (TPS) to protect the vehicle against the high-temperature environment. For the design of these TPS, the transient surface heat flux is an important parameter^1^. On the other hand, the demisability of spacecraft is currently of great concern to the aerospace industry^4^. A common method to investigate the demise process is through plasma wind tunnel experiments. Here, high-enthalpy flow conditions are set for the simulation of the aerothermal loads as occurring on a real re-entry flight. However, usually only steady-state conditions are set, i.e. only one particular point on the vehicle’s flight trajectory is investigated^5^. The High Enthalpy Flow Diagnostics Group (HEFDiG) of the Institute of Space Systems, University of Stuttgart, recently established a procedure that enables the simulation of short segments on a re-entry trajectory by conducting transient plasma wind tunnel experiments^6^. This is of particular importance for demisability investigations. However, this new testing scenario requires a transient surface heat flux measurement under continuous high heating loads. These constraints rule out common steady-state techniques such as water and slug calorimeters, as they cannot capture transient heat fluxes^7,8^. Transient methods like the Cook-Felderman technique and currently known regularization approaches are subject to semi-infinite or adiabatic backside boundary conditions^9–13^. Considering heat fluxes in the order of several 100 kW/m^2^ or higher, the energy in the form of heat quickly accumulates in the sensor material due to the finite thermal capacity of the sensor material and too low backbound heat conduction in semi-infinite bodies. As a result, the sensor’s temperature rise ultimately limits the measurement duration to prevent exceeding its maximum temperature. This means high heat fluxes lead to very short measurement durations. Another requirement for heat flux diagnostics in plasma wind tunnels is the ability to measure the heat flux on a cold copper surface, which serves as the standard reference^14^. This requirement excludes the use of gradient heat flux sensors and Gardon gauges, which are otherwise well-suited for transient and continuous measurements^15,16^. As a result, a measurement technique is missing to accurately determine cold wall surface heat flux over a significantly long test duration, e.g. in the order of minutes. With the new method presented in this work, we close this gap.

The Non-Integer System Identification (NISI) approach offers the capability to measure the transient heat flux with increased accuracy compared to the classical techniques^10^. One crucial limitation of the NISI method, which this work aims to mitigate, is that the wall’s material properties are required to remain constant during the measurement. So far, the NISI approach has been derived for an adiabatic and semi-infinite backside boundary^10,11^. As described above, these backside boundary conditions limit the maximum measurement duration. This work extends the NISI approach by deriving it for a backside boundary condition assuming active cooling, enabling long-duration measurements at continuously high heat fluxes. This novel approach is called Non-Integer System Identification with active cooling (NISIc). With a NISIc sensor, the measurement of the transient surface heat flux onto a cold copper wall in plasma wind tunnels for minute time ranges becomes accessible. Furthermore, another promising application for this technique is in rocket engine combustion chambers, where a similar scenario of heat conduction is seen.

In the first part of this paper, we derive the NISI equation for an actively cooled backside boundary. In the second part, we present a NISIc assessment of simulated data to show that the NISIc approach yields useful results.

## System identification

**Fig. 1:**
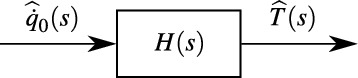
The transfer function in the heat conduction scenario.

The development of the Non-Integer System Identification (NISI) approach aimed to describe a heat conduction system through a simple model equation. This approach is inspired by so-called ARX (Auto-Regression with eXtra inputs) models, which are commonly used in the field of system identification^17^. As illustrated in Fig. 1, the problem of interest is interpreted as a

*system* with an input, the *heat flux*, and an output, the *temperature*. Between the two quantities, some sort of transfer function

1 $$\begin{aligned} H(s) =\frac{\widehat{T}(s)}{\widehat{\dot{q}}_0(s)} \end{aligned}$$

translates the system input (heat flux ${\dot{q}}_0$) to the system output (temperature *T*). Note, that one single heat flux is correlated to one single temperature signal, where both quantities are a function of time. A typical ARX transfer function in the time domain is of the form^17^

2 $$\begin{aligned} \sum _{i=0}^{L} {{\mathfrak {a}}}_i\, D^i\, T(t) = \sum _{i=0}^{M} {{\mathfrak {b}}}_i\, D^i \,\dot{q}_0(t) \qquad \textrm{with} \qquad D^i = \frac{d^i}{dt^i}, {{\mathfrak {a}}}_0 = 1 \end{aligned}$$

with the model parameters ${{\mathfrak {a}}}_i$ and ${{\mathfrak {b}}}_i$ which are to be identified. Such equations meaningfully model a complex physical process as simple mathematical formulations. The transfer function can be found by e.g. sensor calibration. The knowledge of the transfer function eventually enables the determination of the desired heat flux input from the measurement of only one single temporally resolved temperature output. This methodology was used in numerous real applications, e.g.^18–24^.

Without going into further details here, the fundamental characteristic of the system identification approach in a heat conduction system is the merit of including non-integer derivatives, which is the reason why the method is called *non-integer system identification*. Battaglia showed that there is an analytical derivation of a one-dimensional heat conduction problem with a semi-infinite and an adiabatic boundary condition using the approach of solving the problem by a Laplace transformation of the heat equation^10^. The Laplace approach for the adiabatic boundary condition was also shown by Loehle^11^.

In the following the same train of thought is followed with the new boundary condition of a cooled backside. It will turn out that the resulting fundamental equation is the same as for the known problems, which shows again the versatility of this NISI approach.

## Mathematical derivation of the NISIc approach

**Fig. 2:**
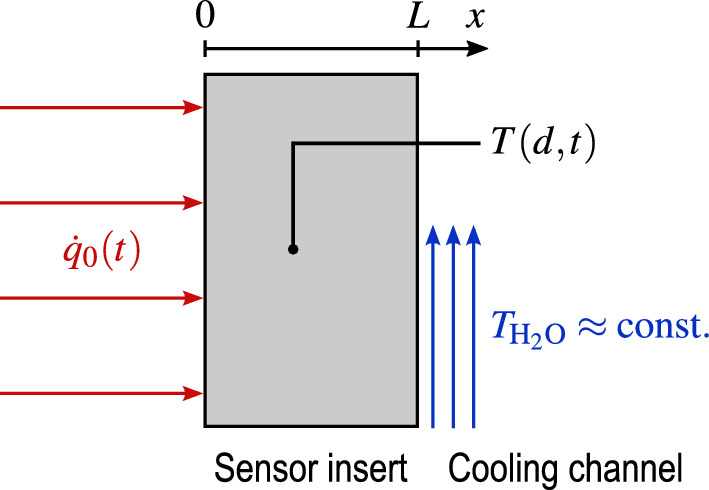
The NISIc heat conduction scenario.

In this section, we derive the NISI equation from the 1D heat equation for an actively cooled backside as depicted in Fig. 2. The cooling of the sensor element is assumed to be realized by a water cooling circuit at its backside. The sensor element itself is made out of copper. This has the advantage of a high thermal conductivity and a useful surface chemistry for testing in chemically reactive flow fields^2^. The crucial assumption for this derivation is that the backside is cooled sufficiently so that we can reasonably well assume a negligible water temperature increase with respect to the temperature difference between the backside and the cooling water. If this assumption is valid and if a constant coolant mass flow rate, hence a constant heat transfer coefficient is established, the heat loss through the back boundary is linear to the backside temperature.

### Assumption of negligible water temperature increase

The heat transfer from the convectively cooled backside into the cooling water is given by^25^

3 $$\begin{aligned} \dot{q}_\textrm{L}(t) = a\,\left( T_\textrm{L}(t) - \overline{T}_\mathrm {H_2O}(t) \right) = a\,\left( \left( T_\textrm{i} + \Delta T_\textrm{L}(t)\right) - \left( T_\mathrm {H_2O,in} + \Delta \overline{T}_\mathrm {H_2O}(t)\right) \right) = a\,\left( \Delta T_\textrm{L}(t) - \Delta \overline{T}_\mathrm {H_2O}(t) \right) \end{aligned}$$

with the convective heat transfer coefficient *a*. The initial backside temperature $T_\textrm{L}(t)$ can be expressed by the sum of the initial backside temperature $T_\textrm{i}$ and the backside’s temperature difference $\Delta T_\textrm{L}(t)$. The average temperature of the cooling water ${\overline{T}}_\mathrm {H_2O}(t)$ can be expressed by the sum of inbound water temperature $T_\mathrm {H_2O,in}$ and the average cooling water difference $\Delta {\overline{T}}_\mathrm {H_2O}(t) = \left( T_\mathrm {H_2O,out}(t)-T_\mathrm {H_2O,in} \right) / 2$, accordingly. The inbound water temperature is assumed to remain constant at the initial temperature, i.e. $T_\mathrm {H_2O,in} = T_\textrm{i}$ due to the fact that the cooling water comes from the immense water reservoir in the laboratory cooling system which includes another cooling circuit to stabilize the water temperature.

For the second boundary condition of the heat conduction problem (Eq. (8c)) we aim for an expression where the heat flux out of the wall is linearly related to the backside’s temperature difference, i.e.

4 $$\dot{q}_\textrm{L}(t) \propto \Delta T_\textrm{L}(t). $$

From Eq. (3) we can see that the approximation in Eq. (4) is correct within 1% if

5 $$\begin{aligned} \Delta T_\textrm{L}(t)> 100\,\Delta {\overline{T}}_\mathrm {H_2O}(t). \end{aligned}$$

This can be realized by providing sufficiently high cooling water mass flow rates $\dot{m}$, which is seen from the classical definition of calorimetric water cooling^7^

6 $$\begin{aligned} \dot{Q}_\mathrm {H_2O}(t) = \dot{q}_\textrm{L}(t)\,A_\textrm{s} = \dot{m}\,c_\mathrm {p,H_2O}\,\Delta {T}_\mathrm {H_2O,total}(t), \end{aligned}$$

where $\dot{m}$ is inversely proportional to the total cooling water temperature increase $\Delta {T}_\mathrm {H_2O,total}(t) = T_\mathrm {H_2O,out}(t)-T_\mathrm {H_2O,in}$, i.e. for a given heat flow $\dot{Q}_\mathrm {H_2O}(t)$, an increase of $\dot{m}$ decreases the temperature difference $\Delta {T}_\mathrm {H_2O,total}(t)$. In Eq. (6), $A_\textrm{s}$ is the cross-sectional area of the solid wall, and $c_\mathrm {p,H_2O}$ is the specific heat capacity of water. The minimum cooling water mass flow rate is found by substitution of Eq. (6) into Eq. (3) and substitution of $\Delta T_\textrm{L}(t)$ with Eq. (5) to get

7 $$\begin{aligned} \begin{aligned} \frac{\dot{m}\,c_\mathrm {p,H_2O}}{A_\textrm{s}}\,\Delta {T}_\mathrm {H_2O,total}(t)&= a\,\left( \Delta T_\textrm{L}(t) - \Delta {\overline{T}}_\mathrm {H_2O}(t) \right)> a\,\left( 100\,\Delta {\overline{T}}_\mathrm {H_2O}(t) - \Delta {\overline{T}}_\mathrm {H_2O}(t) \right) \\ \frac{\dot{m}\,c_\mathrm {p,H_2O}}{A_\textrm{s}}\,2\,\Delta {\overline{T}}_\mathrm {H_2O}(t)&> 99\,a\,\Delta {\overline{T}}_\mathrm {H_2O}(t)\\ \dot{m}&> 49.5\,\frac{A_\textrm{s}\,a}{c_\mathrm {p,H_2O}}. \end{aligned} \end{aligned}$$

Eq. (7) provides a lower limit of the water cooling that is required by the NISIc approach. The heat transfer coefficient *a* can be found from the Nusselt and Reynolds numbers using common correlations^25^. However, its calculation depends on the geometry of the cooling channels. The reasons are that firstly, the utilized correlation depends on the flow direction, i.e. parallel or impinging flow. Secondly, the Reynolds number is a function of flow velocity which is driven by the cross sectional area of the cooling channel. A realistic estimate is a minimum mass flow rate of approximately $20\,$g/s to satisfy Eq. (5) for a NISIc sensor system with a parallel water flow and an insert diameter of about 15 mm. The satisfaction of Eq. (5) means that the assumption that the outbound heat flux at the backside is linear to the backside’s temperature difference is correct to below 1%. The validity of this assumption is required by the second boundary condition (Eq. (8c)) in the heat conduction problem which is given in the next subsection.

### Solution of 1D heat equation for actively cooled back boundary

Consider the 1D heat conduction scenario given in Fig. 2. The corresponding 1D heat conduction problem is given by

8a $$\begin{aligned}&\frac{\partial T(x,t)}{\partial t} = \alpha \frac{\partial ^2 T(x,t)}{\partial x^2} \qquad 0<x<L, \quad t\ge 0 \end{aligned}$$

with the temperature *T*, spatial coordinate *x* and temporal variable *t*, the wall thickness *L*, and the material properties thermal diffusivity $\alpha =\lambda /(\rho \,c_\textrm{p})$, thermal conductivity $\lambda$, density $\rho$, and specific heat capacity $c_\textrm{p}$. The boundary conditions are given by

8b $$\begin{aligned}&-\lambda \frac{\partial T(0,t)}{\partial x} = \dot{q}(0,t) = \dot{q}_0(t) \qquad t\ge 0 \end{aligned}$$

8c $$\begin{aligned}&-\lambda \frac{\partial T(L,t)}{\partial x} = \dot{q}(L,t) = a\,\left( T(L,t) - T_\mathrm {H_2O}\right) \qquad t\ge 0 \end{aligned}$$

and the initial condition by

8d $$\begin{aligned} T(x,0) = T_\mathrm {H_2O} = T_\textrm{i} \qquad 0<x<\infty . \end{aligned}$$

Here, $\dot{q}_0(t)$ is the net surface heat flux, which is absorbed by the wall. $T_\mathrm {H_2O}$ is the temperature of the cooling water. Remember, that the fundamental assumption of this approach is that $T_\mathrm {H_2O}$ increases negligibly compared to the temperature difference $T(L,t) - T_\mathrm {H_2O}$. Therefore, $T_\mathrm {H_2O}$ is considered constant and equal to the initial temperature $T_\textrm{i}$. Consequently, the heat loss through the backside $\dot{q}(L,t)$ is then linear to the backside temperature *T*(*L*, *t*) by the factor of the heat transfer coefficient *a*. Therefore, Eq. (8c) is categorized as a Robin boundary condition. Eq. (8b) is a Neumann boundary condition.

We follow the approach of solving the heat conduction problem using the Laplace transformation. The Laplace transform of Eqs. (8a) to (8d) gives the ordinary differential equation

9a $$\begin{aligned} \frac{\textrm{d}^2 \widehat{T}(x,s)}{\textrm{d} x^2} = \frac{s}{\alpha } \widehat{T}(x,s) \end{aligned}$$

with the Laplace variable *s* and the Laplace transformed boundary and initial conditions

9b $$\begin{aligned}&\frac{\textrm{d} \widehat{T}(0,s)}{\textrm{d} x} = -\frac{1}{\lambda }\,\widehat{\dot{q}}_0(s) \end{aligned}$$

9c $$\begin{aligned}&\frac{\textrm{d} \widehat{T}(L,s)}{\textrm{d} x} = -\frac{k}{\lambda }\,\left( \widehat{T}(L,s)-\widehat{T}_\textrm{i}\right) \end{aligned}$$

9d $$\begin{aligned} \widehat{T}(x,s\rightarrow \infty ) = \widehat{T}_\textrm{i} . \end{aligned}$$

The differential equation (9a) can be solved by the exponential Ansatz

10 $$\begin{aligned} \widehat{T}(x,s) = K_1\,e^{-x \sqrt{\frac{s}{\alpha }}} + K_2\,e^{x \sqrt{\frac{s}{\alpha }}}. \end{aligned}$$

Differentiation of Eq. (10) with respect to *x* gives

11 $$\begin{aligned} \frac{\textrm{d}\widehat{T}(x,s)}{\textrm{d}x} = - \sqrt{\frac{s}{\alpha }}\,K_1\,e^{-x \sqrt{\frac{s}{\alpha }}} + \sqrt{\frac{s}{\alpha }}\,K_2\,e^{x \sqrt{\frac{s}{\alpha }}}. \end{aligned}$$

We find the constants $K_1$ and $K_2$ by applying the Laplace transformed boundary conditions (Eqs. (9b) and (9c)) into Eq. (11) resulting in

12 $$\begin{aligned} \frac{\textrm{d}\widehat{T}(0,s)}{\textrm{d}x}&= -\frac{1}{\lambda }\,\widehat{\dot{q}}_0(s) = - \sqrt{\frac{s}{\alpha }}\,K_1\,+ \sqrt{\frac{s}{\alpha }}\,K_2 \end{aligned}$$

13 $$\begin{aligned} \frac{\textrm{d}\widehat{T}(L,s)}{\textrm{d}x}&= -\frac{k}{\lambda }\,\left( \widehat{T}(L,s) - T_\textrm{i}\right) = - \sqrt{\frac{s}{\alpha }}\,K_1\,e^{-L \sqrt{\frac{s}{\alpha }}} + \sqrt{\frac{s}{\alpha }}\,K_2\,e^{L \sqrt{\frac{s}{\alpha }}} \end{aligned}$$

and from Eq. (12) it follows

14 $$\begin{aligned} K_2 = K_1 - \sqrt{\frac{\alpha }{s}}\,\frac{1}{\lambda }\,\widehat{\dot{q}}_0(s). \end{aligned}$$

Plugging Eq. (14) into Eq. (13) gives

15 $$\begin{aligned} K_1= \sqrt{\frac{\alpha }{s}}\,\frac{1}{\lambda } \frac{ \widehat{\dot{q}}_0(s)\,e^{L \sqrt{\frac{s}{\alpha }}} -k\,\left( \widehat{T}(L,s) - T_\textrm{i}\right) }{e^{L \sqrt{\frac{s}{\alpha }}}-e^{-L \sqrt{\frac{s}{\alpha }}} } \end{aligned}$$

and again with Eq. (14) follows

16 $$\begin{aligned} K_2 = \sqrt{\frac{\alpha }{s}}\,\frac{1}{\lambda } \left( \frac{ \widehat{\dot{q}}_0(s)\,e^{L \sqrt{\frac{s}{\alpha }}} -k\,\left( \widehat{T}(L,s) - T_\textrm{i}\right) }{e^{L \sqrt{\frac{s}{\alpha }}}-e^{-L \sqrt{\frac{s}{\alpha }}} } - \widehat{\dot{q}}_0(s)\right) . \end{aligned}$$

With the found constants $K_1$ and $K_2$ we can reformulate Eq. 10 yielding

17 $$\begin{aligned} \begin{aligned} \widehat{T}(x,s)&= \sqrt{\frac{\alpha }{s}}\,\frac{1}{\lambda } \frac{ \widehat{\dot{q}}_0(s)\,e^{L \sqrt{\frac{s}{\alpha }}} -k\,\left( \widehat{T}(L,s) - T_\textrm{i}\right) }{e^{L \sqrt{\frac{s}{\alpha }}}-e^{-L \sqrt{\frac{s}{\alpha }}} }\,e^{-x \sqrt{\frac{s}{\alpha }}} + \sqrt{\frac{\alpha }{s}}\,\frac{1}{\lambda } \left( \frac{ \widehat{\dot{q}}_0(s)\,e^{L \sqrt{\frac{s}{\alpha }}} -k\,\left( \widehat{T}(L,s) - T_\textrm{i}\right) }{e^{L \sqrt{\frac{s}{\alpha }}}-e^{-L \sqrt{\frac{s}{\alpha }}} } - \widehat{\dot{q}}_0(s)\right) \,e^{x \sqrt{\frac{s}{\alpha }}}\\&= \sqrt{\frac{\alpha }{s}}\,\frac{1}{\lambda }\left( \frac{ \widehat{\dot{q}}_0(s)\,e^{L \sqrt{\frac{s}{\alpha }}} -k\,\left( \widehat{T}(L,s) - T_\textrm{i}\right) }{e^{L \sqrt{\frac{s}{\alpha }}}-e^{-L \sqrt{\frac{s}{\alpha }}} }\,\left( e^{-x \sqrt{\frac{s}{\alpha }}}+e^{x \sqrt{\frac{s}{\alpha }}}\right) - \widehat{\dot{q}}_0(s)\,e^{x \sqrt{\frac{s}{\alpha }}} \right) \\&= \sqrt{\frac{\alpha }{s}}\,\frac{1}{\lambda }\left( \left( \widehat{\dot{q}}_0(s)\,e^{L \sqrt{\frac{s}{\alpha }}} -k\,\left( \widehat{T}(L,s) - T_\textrm{i}\right) \right) \,\frac{\cosh \left( x \sqrt{\frac{s}{\alpha }}\right) }{\sinh \left( L \sqrt{\frac{s}{\alpha }}\right) } - \widehat{\dot{q}}_0(s)\,e^{x \sqrt{\frac{s}{\alpha }}} \right) \\&= \sqrt{\frac{\alpha }{s}}\,\frac{1}{\lambda }\left( \widehat{\dot{q}}_0(s)\,\left( e^{L \sqrt{\frac{s}{\alpha }}}\, \frac{\cosh \left( x \sqrt{\frac{s}{\alpha }}\right) }{\sinh \left( L \sqrt{\frac{s}{\alpha }}\right) } - e^{x \sqrt{\frac{s}{\alpha }}}\right) -k\,\left( \widehat{T}(L,s) - T_\textrm{i}\right) \,\frac{\cosh \left( x \sqrt{\frac{s}{\alpha }}\right) }{\sinh \left( L \sqrt{\frac{s}{\alpha }}\right) } \right) \end{aligned} \end{aligned}$$

with^26^

18 $$\begin{aligned}&\sinh (z) = \frac{e^z-e^{-z}}{2} = \frac{e^{2z}-1}{2e^z} \end{aligned}$$

19 $$\begin{aligned}&\cosh (z) = \frac{e^z+e^{-z}}{2}= \frac{e^{2z}+1}{2e^z}. \end{aligned}$$

Equation 17 describes the temperature rise inside the material at a position *x* depending on the temperature at the backside $\hat{T}(L,s)$ and the thermophyiscal properties of the material. A generalized solution independent from $\widehat{T}(L,s)$ is found if we consider Eq. (17) at the location $x=L$ and solve then for $\widehat{T}(L,s)$.

20 $$\begin{aligned} \begin{aligned}&\widehat{T}(L,s) = \sqrt{\frac{\alpha }{s}}\,\frac{1}{\lambda }\left( \widehat{\dot{q}}_0(s)\,\left( e^{L \sqrt{\frac{s}{\alpha }}}\, \frac{\cosh \left( L \sqrt{\frac{s}{\alpha }}\right) }{\sinh \left( L \sqrt{\frac{s}{\alpha }}\right) } - e^{L \sqrt{\frac{s}{\alpha }}}\right) -k\,\left( \widehat{T}(L,s) - T_\textrm{i}\right) \,\frac{\cosh \left( L \sqrt{\frac{s}{\alpha }}\right) }{\sinh \left( L \sqrt{\frac{s}{\alpha }}\right) } \right) \\&\widehat{T}(L,s)\left( \lambda \,\sqrt{\frac{s}{\alpha }} + k\, \frac{\cosh \left( L \sqrt{\frac{s}{\alpha }}\right) }{\sinh \left( L \sqrt{\frac{s}{\alpha }}\right) } \right) = \widehat{\dot{q}}_0(s)\,\left( e^{L \sqrt{\frac{s}{\alpha }}}\, \frac{\cosh \left( L \sqrt{\frac{s}{\alpha }}\right) }{\sinh \left( L \sqrt{\frac{s}{\alpha }}\right) } - e^{L \sqrt{\frac{s}{\alpha }}}\right) +k\, T_\textrm{i} \,\frac{\cosh \left( L \sqrt{\frac{s}{\alpha }}\right) }{\sinh \left( L \sqrt{\frac{s}{\alpha }}\right) }\\&\widehat{T}(L,s)= \frac{\widehat{\dot{q}}_0(s)\,\left( e^{L \sqrt{\frac{s}{\alpha }}}\, \cosh \left( L \sqrt{\frac{s}{\alpha }}\right) - e^{L \sqrt{\frac{s}{\alpha }}}\,\sinh \left( L \sqrt{\frac{s}{\alpha }}\right) \right) +k\, T_\textrm{i} \,\cosh \left( L \sqrt{\frac{s}{\alpha }}\right) }{\lambda \,\sqrt{\frac{s}{\alpha }}\,\sinh \left( L \sqrt{\frac{s}{\alpha }}\right) + k\,\cosh \left( L \sqrt{\frac{s}{\alpha }}\right) } \\&\widehat{T}(L,s)= \frac{\widehat{\dot{q}}_0(s) +k\, T_\textrm{i} \,\cosh \left( L \sqrt{\frac{s}{\alpha }}\right) }{\lambda \,\sqrt{\frac{s}{\alpha }}\,\sinh \left( L \sqrt{\frac{s}{\alpha }}\right) + k\,\cosh \left( L \sqrt{\frac{s}{\alpha }}\right) } . \end{aligned} \end{aligned}$$

Eq. (20) applied to Eq. (17) results in the general solution

21 $$\begin{aligned} \widehat{T}(x,s)= \sqrt{\frac{\alpha }{s}}\,\frac{1}{\lambda }\left( \widehat{\dot{q}}_0(s)\,\left( e^{L \sqrt{\frac{s}{\alpha }}}\, \frac{\cosh \left( x \sqrt{\frac{s}{\alpha }}\right) }{\sinh \left( L \sqrt{\frac{s}{\alpha }}\right) } - e^{x \sqrt{\frac{s}{\alpha }}}\right) -k\,\left( \frac{\widehat{\dot{q}}_0(s) +k\, T_\textrm{i} \,\cosh \left( L \sqrt{\frac{s}{\alpha }}\right) }{\lambda \,\sqrt{\frac{s}{\alpha }}\,\sinh \left( L \sqrt{\frac{s}{\alpha }}\right) + k\,\cosh \left( L \sqrt{\frac{s}{\alpha }}\right) } - T_\textrm{i}\right) \,\frac{\cosh \left( x \sqrt{\frac{s}{\alpha }}\right) }{\sinh \left( L \sqrt{\frac{s}{\alpha }}\right) } \right) . \end{aligned}$$

### Derivation of the transfer function through series expansion

From the solution of the heat conduction problem with a cooled backside boundary condition (Eq. (21)), the transfer function can be formulated. For simplification we consider from now on the temperature difference to the initial temperature instead of an absolute temperature. This corresponds to setting $T_\textrm{i} = 0$. This allows to factor out $\widehat{\dot{q}}_0(s)$ on the right side of Eq. (21) and to formulate the transfer function

22 $$\begin{aligned} \begin{aligned}&H(x,s) = \frac{\widehat{T}(x,s)}{\widehat{\dot{q}}_0(s)} = \sqrt{\frac{\alpha }{s}}\,\frac{1}{\lambda } \left( \left( e^{L \sqrt{\frac{s}{\alpha }}} - \frac{k}{ \lambda \,\sqrt{\frac{s}{\alpha }}\,\sinh \left( L \sqrt{\frac{s}{\alpha }}\right) + k\,\cosh \left( L \sqrt{\frac{s}{\alpha }}\right) }\right) \, \frac{\cosh \left( x \sqrt{\frac{s}{\alpha }}\right) }{\sinh \left( L \sqrt{\frac{s}{\alpha }}\right) } - e^{x \sqrt{\frac{s}{\alpha }}} \right) \\&= \frac{\left( \left( \sinh \left( L \sqrt{\frac{s}{\alpha }}\right) + \sqrt{\frac{\alpha }{s}}\,\frac{k}{\lambda }\,\cosh \left( L \sqrt{\frac{s}{\alpha }}\right) \right) e^{L \sqrt{\frac{s}{\alpha }}}-\sqrt{\frac{\alpha }{s}}\,\frac{k}{\lambda }\right) \,\cosh \left( x \sqrt{\frac{s}{\alpha }}\right) }{\left( \lambda \,\sqrt{\frac{s}{\alpha }}\,\sinh \left( L \sqrt{\frac{s}{\alpha }}\right) + k\,\cosh \left( L \sqrt{\frac{s}{\alpha }}\right) \right) \sinh \left( L \sqrt{\frac{s}{\alpha }}\right) } \\&\hspace{6.7cm} - \frac{e^{x \sqrt{\frac{s}{\alpha }}}\,\sinh \left( L \sqrt{\frac{s}{\alpha }}\right) \, \left( \sinh \left( L \sqrt{\frac{s}{\alpha }}\right) + \sqrt{\frac{\alpha }{s}}\,\frac{k}{\lambda }\,\cosh \left( L \sqrt{\frac{s}{\alpha }}\right) \right) }{\left( \lambda \,\sqrt{\frac{s}{\alpha }}\,\sinh \left( L \sqrt{\frac{s}{\alpha }}\right) + k\,\cosh \left( L \sqrt{\frac{s}{\alpha }}\right) \right) \sinh \left( L \sqrt{\frac{s}{\alpha }}\right) }\\&= \frac{\left( \sinh \left( L \sqrt{\frac{s}{\alpha }}\right) + \sqrt{\frac{\alpha }{s}}\,\frac{k}{\lambda }\,\cosh \left( L \sqrt{\frac{s}{\alpha }}\right) \right) \left( e^{L \sqrt{\frac{s}{\alpha }}}\,\cosh \left( x \sqrt{\frac{s}{\alpha }}\right) - e^{x \sqrt{\frac{s}{\alpha }}}\, \sinh \left( L \sqrt{\frac{s}{\alpha }}\right) \right) -\sqrt{\frac{\alpha }{s}}\,\frac{k}{\lambda }\,\cosh \left( x \sqrt{\frac{s}{\alpha }}\right) }{\left( \lambda \,\sqrt{\frac{s}{\alpha }}\,\sinh \left( L \sqrt{\frac{s}{\alpha }}\right) + k\,\cosh \left( L \sqrt{\frac{s}{\alpha }}\right) \right) \sinh \left( L \sqrt{\frac{s}{\alpha }}\right) } . \end{aligned} \end{aligned}$$

We develop the hyperbolic and the exponential functions with^26^

23 $$\begin{aligned}&\sinh (z) = \sum _{n=0}^{\infty }\frac{z^{2n+1}}{(2n+1)!} \end{aligned}$$

24 $$\begin{aligned}&\cosh (z) = \sum _{n=0}^{\infty }\frac{z^{2n}}{(2n)!} \end{aligned}$$

25 $$\begin{aligned}&e^z = \sum _{n=0}^{\infty }\frac{z^{n}}{n!} \end{aligned}$$

to obtain

26 $$\begin{aligned} H(x,s) = \frac{\left( \sum \limits _{n=0}^{\infty }\hspace{-1mm}\frac{\left( L \sqrt{\frac{s}{\alpha }}\right) ^{2n+1}}{(2n+1)!} + \sqrt{\frac{\alpha }{s}}\frac{k}{\lambda }\sum \limits _{n=0}^{\infty }\hspace{-1mm}\frac{\left( L \sqrt{\frac{s}{\alpha }}\right) ^{2n}}{(2n)!}\right) \hspace{-1mm} \left( \sum \limits _{n=0}^{\infty }\hspace{-1mm}\frac{\left( L \sqrt{\frac{s}{\alpha }}\right) ^{n}}{n!}\sum \limits _{n=0}^{\infty } \hspace{-1mm}\frac{\left( x \sqrt{\frac{s}{\alpha }}\right) ^{2n}}{(2n)!} - \sum \limits _{n=0}^{\infty }\hspace{-1mm}\frac{\left( x \sqrt{\frac{s}{\alpha }}\right) ^{n}}{n!} \sum \limits _{n=0}^{\infty }\hspace{-1mm}\frac{\left( L \sqrt{\frac{s}{\alpha }}\right) ^{2n+1}}{(2n+1)!} \right) -\sqrt{\frac{\alpha }{s}}\frac{k}{\lambda }\sum \limits _{n=0}^{\infty }\hspace{-1mm}\frac{\left( x \sqrt{\frac{s}{\alpha }}\right) ^{2n}}{(2n)!}}{\left( \lambda \sqrt{\frac{s}{\alpha }}\sum \limits _{n=0}^{\infty }\hspace{-1mm}\frac{\left( L \sqrt{\frac{s}{\alpha }}\right) ^{2n+1}}{(2n+1)!} + k\sum \limits _{n=0}^{\infty }\hspace{-1mm}\frac{\left( L \sqrt{\frac{s}{\alpha }}\right) ^{2n}}{(2n)!}\right) \sum \limits _{n=0}^{\infty }\hspace{-1mm}\frac{\left( L \sqrt{\frac{s}{\alpha }}\right) ^{2n+1}}{(2n+1)!}}. \end{aligned}$$

If we expand the sum terms, we can see, that at least one term to each power of $s^{(n-1)/2}$ with $n \in \mathbb {N}_0$ exists in the numerator. The same goes for the denominator with the exception of $s^{-1/2}$ and $s^0$. We can combine the respective prefactors into the coefficients $\alpha _n$ and $\beta _n$. This allows to bring Eq. (26) into a general form

27 $$\begin{aligned} H(x,s) = \frac{\sum _{n=0}^{\infty }\beta _n \, s^{(n-1)/2}}{\sum _{n=0}^{\infty }\alpha _n\,s^{(n+1)/2}}. \end{aligned}$$

Transforming Eq. (27) into the time domain results in

28 $$\begin{aligned} \sum _{n=0}^{\infty } \alpha _n\, D^{(n+1)/2}\, T(x,t) = \sum _{n=0}^{\infty } \beta _n\, D^{(n-1)/2} \,\dot{q}_0(t). \end{aligned}$$

Eq. (27) is very similar to the solutions of Battaglia and Loehle for a semi-infinite or adiabatic back boundary condition respectively^10,11^. Battaglia noticed that these solutions differ from the standard ARX model equation (Eq. (2)) mainly by the half-order derivatives. On this basis, he enhanced the ARX model equation for the application in heat conduction problems to include these half-order derivatives^10^. This eventually resulted in the NISI equation

29 $$\begin{aligned} \sum _{n=M_0}^{M} \alpha _n\, D^{n/2}\, T(t) = \sum _{n=L_0}^{L} \beta _n\, D^{n/2} \,\dot{q}_0(t) \end{aligned}$$

which is commonly used for the modeling of heat conduction problems^10,11^. Here, $L_0$, $M_0$, *L*, and $M\in \mathbb {Z}$ are the variable lower and upper limits of the summations. The temperature *T*(*t*) means the temperature recorded by the thermocouple at its distinct measurement location. The model parameters $\alpha _n$ and $\beta _n$ are to be identified, which is usually done by a non-destructive calibration of the actual sensor system. With this, Eq. (29) allows for the determination of the surface heat flux from a single temperature measurement in the sensor wall.

In conclusion, we derived the classical NISI equation (Eq. (29)) from the heat conduction problem considering the new back boundary condition of an actively cooled wall. This shows again the versatility of the NISI approach. The equation holds for semi-infinite, adiabatic and now cooled back boundary condition.

## NISIc application using simulated data

### Thermal model

**Fig. 3:**
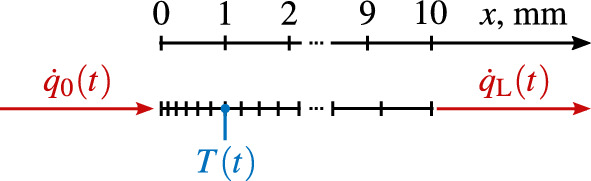
Schematic of the thermal model.

We apply the NISIc approach to a numerical finite elements (FE) simulation of the NISIc approach. The assessment of the simulated data allows us to prove the concept of the NISIc method, i.e. that a sensor with an actively cooled backside behaves linearly to the input heat flux. The heat transfer simulation was conducted with the commercial software *COMSOL Multiphysics*. A schematic of the thermal model is shown in Fig. 3. We modeled a one-dimensional, $10\,$mm long body with 23 elements. The last element at the backside is nine times the length of the first surface element with a linear length increase in between. The material parameters were chosen according to copper, i.e. specific heat capacity $c_p=385\,$J/(kg K), density $\rho = 8932\,$kg/m$^3$, and thermal conductivity $\lambda = 390$ W/(m K)^27^. The front side is subject to the surface heat flux boundary condition (Eq. (8b)) and the opposing side is subject to the backside boundary condition (Eq. (8c)). The water temperature is held at the initial temperature, i.e. $T_\mathrm {H_2O} = T_\textrm{i}$. We chose a heat transfer coefficient of $a = 18000$ W/(m$^2$ K), which corresponds to a water cooling mass flow rate of $\dot{m}=150$ g/s under parallel, turbulent flow in a channel with a cross-sectional area of $3.6\cdot 10^{-5}$ m$^2$. The actual temperature readout spot (*T*(*t*) in Fig. 3), which represents the thermocouple measurement in a real sensor, is placed at a depth of $1\,$mm beneath the heated side. The surface heat flux ($\dot{q}_0(t)$ in Fig. 3) is defined manually to imitate an actual sensor calibration and measurement. This single temperature signal and the surface heat flux are the relevant inputs for the NISI approach.

### NISIc Methodology for heat flux measurements

**Fig. 4:**
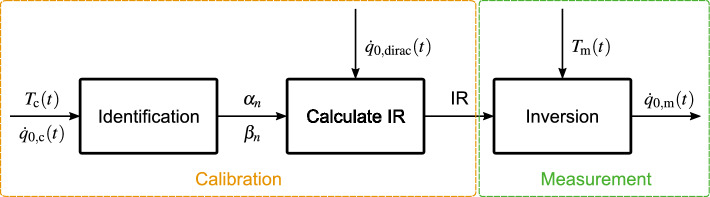
Schematic of the Non-Integer System Identification with active cooling (NISIc) methodology.

The NISIc measurement sequence is sketched in Fig. 4. It consists of the calibration, indicated by the yellow box, and the subsequent application of the sensor system in a measurement scenario, indicated by the green box in Fig. 4.

Although the results presented in this paper are based on simulated data generated using a thermal model, the NISIc measurement sequence is explained in the context of a real-world application. The schematic of a typical NISIc sensor system is shown in Figs. 2 and 3. In the calibration stage, a well-known surface heat flux is applied to the sensor surface and the resulting temperature signal is recorded. A diode laser serves as the heat source, modulating the heat flux into pulses, ideally with varying pulse durations^28^. This is a common approach in system identification^17^. In reality, it is important that the actual sensor of interest is calibrated so that no difference in mounting, material, etc. has to be considered. The reason is that the thermal response at the measurement location is highly sensitive to these parameters. To address this challenge, the NISI methodology, i.e. a system identification approach based on non-destructive sensor system calibration, offers a significant advantage: it eliminates the need for prior knowledge of material properties, detailed sensor geometry, or precise thermocouple positioning. The only required inputs are the emissivity of the sensor surface at the calibration laser wavelength and the power density of the laser spot. These two parameters, along with the uncertainty in temperature measurement, are the sole contributors to the overall measurement uncertainty. All three propagate linearly into the uncertainty of the resulting heat flux signal. Uncertainties arising from the model equation itself are not considered.

The calibration data needed eventually is the impulse response of the system of interest. Its direct determination would require a Dirac heat impulse, which firstly cannot be realized with a laser and secondly, the resulting temperature response is too weak to be usefully measured. The calibration with various pulses emulates the response of the system to many different pulse intervals and thus frequencies in order to cover the range of response frequencies required for the system to be calibrated for the time frame of interest.

The pulsed calibration heat flux $\dot{q}_\textrm{0,c}(t)$ and the temperature reading at the readout spot $T_\textrm{c}(t)$ obtained in the calibration measurement are input to the *Identification* step (see Fig. 4). Here, $T_\textrm{c}(t)$ means the temperature difference from the initial temperature. In the *Identification* step, the model parameters $\alpha _n$ and $\beta _n$ in Eq. (29) are identified using a least squares approach. The found parameters are then used in a second step to calculate the system’s impulse response from^10^

30 $$\begin{aligned} T({k}) = \frac{1}{{{\mathfrak {a}}}_1}\left( \sum _{n=L_0}^{L} \beta _n\, D^{n/2} \,\dot{q}_0({k}) - \sum _{j=2}^{k} {\mathfrak a}_j\,T({k-j+1}) \right) , \quad \textrm{with} \quad {\mathfrak a}_j = \sum _{n=M_0}^{M} \frac{\alpha _n}{(\Delta t)^{n/2}} (-1)^j \left( {\begin{array}{c}{n/2}\\ j\end{array}}\right) \quad \textrm{and} \quad 0< j <k \end{aligned}$$

by using a Dirac heat impulse $\dot{q}_\textrm{0,dirac}(t)$ of magnitude $1\,$W/m$^2$ and the length of one time step as input for the heat flux vector. The parameters ${{\mathfrak {a}}_j}$ in (30) are a new set of parameters found from the NISI parameters $\alpha _n$.

The impulse response is required for the application of the calibrated sensor system in the measurement scenario. In the *Inversion* step (see Fig. 4), the heat flux $\dot{q}_\textrm{0,m}(t)$ is determined from the impulse response (IR) and the measurement temperature $T_\textrm{m}(t)$. For this, we use the *Sequential Function Estimation* algorithm by Beck^12^, where the so-called future time parameter serves as a regularizaton parameter acting similarly to a filter.

### Results

To demonstrate the NISIc procedure for accurate heat flux determination, we execute the full NISIc sequence with simulated data sets. This means that we first use the thermal model to generate a calibration data set, i.e. apply a pulsed surface heat flux $\dot{q}_\textrm{0,c}(t)$ and extract the temperature $T_\textrm{c}(t)$ at the readout spot as specified in the *Thermal Model* subsection. This numerical data is input to the *Identification* step to find the NISI parameters, which subsequently enable the impulse response calculation. To simulate the measurement scenario, we generate a second data set using the thermal model, where we apply a temporal Gauss shaped heat flux signal $\dot{q}_\textrm{0,input}(t)$ to the surface. The temperature readout in this simulation serves as the measurement temperature $T_\textrm{m}(t)$ for the *Inversion* step (see Fig. 4). The output of this step is the heat flux determined with NISIc $\dot{q}_\textrm{0,m}(t)$.

**Fig. 5:**
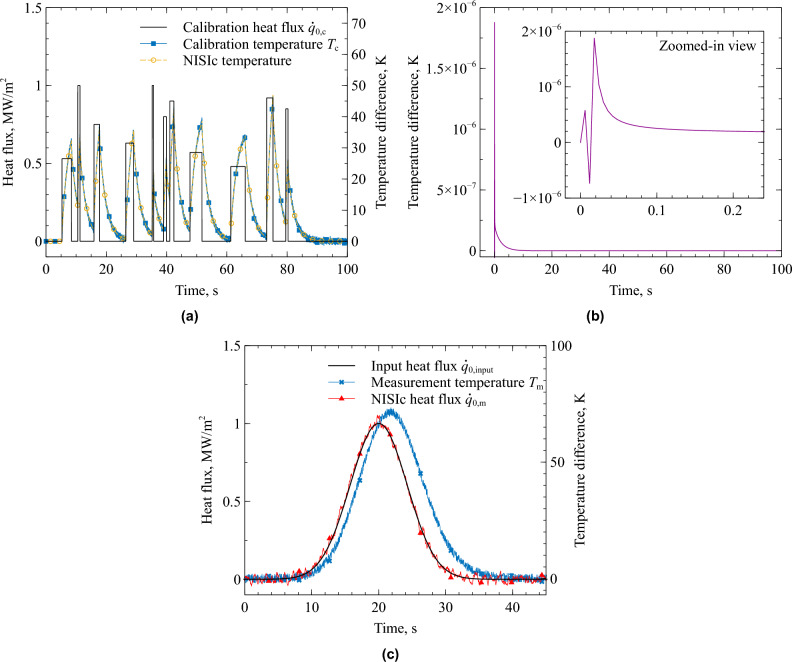
Results of the NISIc analysis of the simulated data; calibration data set (a), resulting impulse response (b), and heat flux determination case (c).

The results of the NISIc assessment of the simulated data are presented in Fig. 5. The simulated calibration data set is shown in Fig. 5a .

The calibration heat flux $\dot{q}_\textrm{0,c}(t)$ is shown in black and the calibration temperature $T_\textrm{c}(t)$ is shown in blue. To simulate a realistic measurement, random noise with a peak-to-peak amplitude of $\pm 2\,$K was added to the numerically calculated calibration temperature data. The noisy temperature data was then filtered with a common FIR filter to arrive at the blue curve shown in Fig. 5a . This calibration temperature data and the calibration heat flux are used for the identification of the NISI parameters $\alpha _n$ and $\beta _n$. It can be shown that the identified parameter set represents the heat conduction process in a useful manner by calculating the temperature response to the calibration heat flux using the identified parameter set and Eq. (30). The resulting NISIc temperature shown in yellow in Fig. 5a matches the blue calibration temperature perfectly.

The impulse response, which is given in Fig. 5b , is found as described in the previous subsection. It can be seen that the impulse response drops to 0 after around $10\,$s. Any heat that is introduced to the surface is carried out of the system after that time, i.e. the heat does not accumulate in the copper piece. This means this sensor can be operated without any time-limitation provided that a certain heat flux threshold is not exceeded. This is the key feature of a NISIc sensor system. Exemplarily for the heat conduction scenario of the presented thermal model and for a maximum temperature at the sensor surface of $100^{\circ }$C, the maximum steady-state heat flux is 1 MW/m$^2$. Reducing the length of the sensor insert to $5\,$mm and for a maximum temperature of $500^{\circ }$C, would push the maximum heat flux above 7 MW/m$^2$.

There is a strong oscillation observable in the impulse response in Fig. 5b shortly after $0\,$s. Such oscillations are often observed in impulse responses that are determined using the NISI method. However, they do not compromise the quality of the eventual heat flux inversion.

The results in Fig. 5c prove that the NISIc approach yields accurate measurement results. The data set for the measurement scenario was generated with the thermal model. The input heat flux $\dot{q}_\textrm{0,input}(t)$, shown in black, was applied at the heat flux boundary (Eq. (8b)). In a real application, this heat flux is unknown and shall be determined using the NISIc approach. However, since it is known in this case, it enables a direct comparison between the heat flux determined by the NISIc method and the actual input. The blue measurement temperature $T_\textrm{m}(t)$ was extracted from the same readout spot as in the calibration. Noise with a peak-to-peak amplitude of $\pm 2\,$K was added to the measurement temperature. The noisy measurement temperature $T_\textrm{m}(t)$ and the impulse response IR, which is shown in Fig. 5b , are the input to the *Inversion* step to determine the NISIc heat flux $\dot{q}_\textrm{0,m}(t)$. Here, we set a future time parameter of $0.5\,$s for regularization in the *Sequential Function Estimation* algorithm^12^. The resulting NISIc heat flux, i.e. the inversely determined heat flux using NISIc, is given as the red curve in Fig. 5c . It can be seen that the NISIc heat flux matches the input heat flux perfectly. This shows that the NISIc approach is a useful tool for long-duration heat flux measurements. The underlying assumption of the backside boundary condition according to Eq. (8c) proves therefore meaningful because the resulting heat conduction scenario is linear.

## Conclusion

In this paper we derived the classical NISI equation (Eq. (29)) from the heat equation for an actively cooled back boundary condition. The crucial assumption for this is that the cooling water temperature increases negligibly compared to the temperature difference between backside and water. We provide an assessment of a minimum water mass flow rate to reasonably justify this assumption. Finally, we apply this new NISIc approach to data from a thermal simulation. The NISIc approach produces an excellent agreement between the determined heat flux and the known input. This proves the underlying concept of the NISIc approach. In conclusion, this paper provides the community with a new method to measure the transient surface heat flux without any time limitation.

## Data Availability

The datasets generated during and/or analyzed during the current study are available from the corresponding author on reasonable request.
